# Sex-Specific Differences in Related Indicators of Blood Pressure in School-Age Children With Overweight and Obesity: A Cross-Sectional Study

**DOI:** 10.3389/fped.2021.674504

**Published:** 2021-08-05

**Authors:** Hongmei He, Shujun Yang, Na Qiu, Ling Qiao, Yong Ding, Jiajia Luo, Yuan Li, Zengyou Luo, Yingsa Huang, Huishen Pang, Shaoping Ji, Lu Zhang, Xiangqian Guo

**Affiliations:** ^1^Department of Preventive Medicine, School of Basic Medical Sciences, Henan University, Kaifeng, China; ^2^Department of Pediatrics, First Clinical College, Harbin Medical University, Harbin, China; ^3^Department of Biochemistry and Molecular Biology, School of Basic Medical Science, Henan University, Kaifeng, China; ^4^Kaifeng Key Laboratory of Cell Signal Transduction, Henan Provincial Engineering Center for Tumor Molecular Medicine, Kaifeng, China; ^5^Bioinformatics Center, Institute of Biomedical Informatics, Henan University, Kaifeng, China

**Keywords:** pediatric obesity, blood pressure, arterial pressure, sex, China

## Abstract

**Objective:** The objective of this study is to further explore the difference between elevated blood pressure (EBP), elevated pulse pressure (EPP), and elevated mean arterial pressure (EMAP) and obesity in Chinese school-age children by sex.

**Methods:** We performed a cross-sectional study of 935 children between 7 and 12 years old. Overweight and obesity were defined by body mass index and body composition. The multivariate logistic regression and the adjusted population attributable risk were used to assess the effects of obesity on pre-EBP/EBP, EPP, and EMAP. The interactions were used to identify the modification of obese on the relationship between related indicators of blood pressure and height or age.

**Results:** The average age of the children included in the study was 10. Boys with overweight and obesity had higher pre-EBP/EBP, EPP, and EMAP (*p* < 0.05). The multivariate logistic regression analysis showed that overweight and obesity had a greater impact on BP and MAP than PP, especially in boys [odds ratio (OR) > 1]. Pre-EBP/EBP in 79% of boys and 76% of girls could be attributable to the visceral fat level. The interaction between BP, PP, MAP, and height or age was modestly increased in children with overweight and obesity, especially in boys.

**Conclusions:** Independent of age and height, obesity not only increases blood pressure, it also increases mean arterial pressure and pulse pressure, and this effect is more pronounced in boys.

## Introduction

Previous pathophysiological and epidemiological evidence indicates that childhood hypertension is associated with essential hypertension in adults and harmful lifelong cardiovascular events (1–3). Obesity is a major risk factor for elevated blood pressure in children and adolescents (4). Furthermore, the impact of being overweight or obese on elevated blood pressure gradually increased in Chinese children from 2005 to 2014 (5). As there are gender differences in the prevalence and influencing factors for obesity in children, it is necessary to explore the effect of obesity on blood pressure on this same basis. Blood pressure (BP) is usually assessed as systolic blood pressure (SBP) and diastolic blood pressure (DBP). Alternative decomposition into pulse pressure (PP) and mean arterial pressure (MAP) localizes the changes to large and small arteries, respectively, and predicts incident cardiovascular events (6). PP was calculated as SBP minus DBP. The increase in PP that occurs with advancing age is predominantly due to reduced arterial distensibility leading to decreased aortic compliance, which is the main risk factor for coronary heart disease and stroke (7). MAP is an important blood pressure index that comprehensively considers SBP and DBP together, and is calculated as follows: MAP = 1/3 × SBP (mmHg) + 2/3 × DBP (mmHg) (8). MAP represents the steady flow of blood through the aorta and its arteries. Also, MAP is considered a surrogate marker for vascular stiffness resulting from loss of vascular elasticity (9).

Body mass index (BMI) is the standard diagnostic tool to assign overweight and obesity in children and adolescents, but BMI cannot determine the location of fat accumulation. Alternative measurements such as dual-energy x-ray absorptiometry (DEXA) and skin fold thickness may be more precise, but are far more complex to use. Bioelectrical impedance analysis (BIA) is extensively used in epidemiological settings to evaluate body composition, which is non-invasive, low cost, and reliable (10). Therefore, the combination of BMI and BIA can accurately determine the characteristics of childhood obesity.

Here, we used a cross-sectional study to investigate the sex-specific differences in the association between elevated blood pressure (EBP), elevated pulse pressure (EPP), elevated mean arterial pressure (EMAP), and BMI and body composition in Chinese school-age children.

## Materials and Methods

### Study Design and Population

This cross-sectional study was conducted from October to November 2019 in Kaifeng, Henan Province. The sample size was calculated using the case–control study formula,

$$n=\frac{{\left(Z_{\alpha }\sqrt{2\bar{p}\bar{q}}+Z_{\beta }\sqrt{p_{0}q_{0}+p_{1}q_{1}}\right)}^{2}}{{\left(p_{1}-p_{0}\right)}^{2}}$$

*α* = 0.05, *β* = 0.20, *OR* (Odds ratio) = 2, the proportion of EBP in children 9.8% (*P*_0_ = 9.8%) (11), and the case group and the control group with the same sample size. Therefore, the study sample size of 576 children is adequate.

Five primary schools from rural and urban areas were selected from 33 primary schools by cluster sampling. According to the data of the school's routine physical examination last year, 935 school-age children were selected between 7 and 12 years old by simple random sampling, namely, 307 children of normal weight, 228 with overweight, and 400 with obesity. The sampling procedure is shown in Supplementary Figure 1.

A set of standardized questionnaires for students and parents assessed demographic characteristics, lifestyle, diet, learning and social skills, family environment, mother's pregnancy and feeding patterns during infancy, and parents' attitudes toward health knowledge and behavior. Under the guidance of the investigator, the students filled out a questionnaire at school. Students took the parents' questionnaire home and gave it to their parents to be filled in. After the parents' questionnaires were collected, the investigator would call the students' parents if there were any questions. The anthropometric measurements (weight, height, blood pressure, and pulse) and body composition indicators (body fat mass, muscle mass, body fat percentage, visceral fat area, etc.) were measured by trained professionals. Written informed consent was obtained from parents or guardians. The study was approved by the Ethics Committee of Henan University.

### Measurements

Height and weight were measured by using standard methods with a metric scale to the nearest 0.1 cm and 0.1 kg with school-aged children wearing light clothing and no shoes. Blood pressure was measured in triplicate at 30-s intervals by using an electronic sphygmomanometer (Omron HEM-7136) and the mean was used for analysis. According to the size of the child's upper arm circumference, the adult or the child cuff was selected. Body fat, muscle mass, protein mass, body fat percentage, visceral fat area, and visceral fat level were measured by using a bioelectrical impedance analyzer (JAWON ioi353). BMI was calculated as weight in kilograms divided by the square of height in meters.

### Definitions

In this study, pre-EBP and EBP were defined as SBP and/or DBP ≥ age-, sex-, and height-specific 90th percentile and 95th percentile, according to the blood pressure cutoff for Chinese children at 7–17 years of age (12). Overweight and obesity were defined using the sex- and age-specific BMI criteria, that is, BMI percentile at least 85th and 95th, respectively (13). Because there is no unified diagnostic standard, elevated pulse pressure (EPP) and elevated mean arterial pressure (EMAP) were defined as PP and MAP ≥90th percentile of data from this study. For boys aged 6–18 years, body fat percentage (FAT%) ≥20, 25, and 30% were mild, moderate, and severe obesity, respectively. For girls aged 6–14 years, FAT% ≥ 25, 30, and 35% were mild, moderate, and severe obesity, respectively (14).

### Statistical Analysis

Categorical variables were represented with frequencies (%). Continuous variables were represented as mean ± standard deviation or median (interquartile range) according to whether the continuous variable exhibited homogeneity of variance and normal distribution. Mann–Whitney Wilcoxon and Student's *t*-tests were used to compare differences among the categories for continuous data. Pearson's chi-square, Fisher's exact, and chi-square tests of linear trend were used to compare differences among the category data. Multivariate logistic regression analysis was used to determine the independent effect of overweight and obesity on the risk of EBP, EPP, and EMAP with the adjustment of age, setting, height, and heart rate. The population attribution risk (PAR) was obtained using the formula defined by Bruzzi et al. (15), $PAR\%=\left[1-\overset{j}{\underset{i=0}{\sum }}\frac{P_{j}}{R_{j}}\right]\times 100$, which was suitable for case–control studies. In the formula, *P*_j_ = *D*_j_/*D, D* is the total number of cases; stratum *j*, namely, stratum 1, 2, … *j* of the exposure factor level; *D*_j_ is the number of cases in the stratum *j*; *P*_j_ is the ratio of the number of cases in the stratum *j* to the total number of cases; *R*_j_ is the odds ratio in the stratum *j* compared with stratum 1 adjusting for confounding factors.

The correlations between SBP, DBP, PP, and MAP and BMI, FAT%, and visceral fat area were statistically evaluated as continuous measures, using Spearman's rank correlation of a non-normal distribution or Pearson's correlation test. The height–obesity and age–obesity interactions were used to identify the modification of obesity on the relationship between blood pressure and height or age, respectively, by the presence of dichotomous BMI obesity status. The tendencies of modification were reported as mean with standard error, and the means of the normal BMI group and the overweight and obesity group were compared by *t*-test.

The SPSS 26.0 (IBM, Armonk, NY, USA) and GraphPad Prism 6.0 (San Diego, California, USA) were used to perform the statistical analysis in this study. A two-tailed *p* < 0.05 was defined as statistically significant.

## Results

### Characteristics of Participants

A total of 935 school-age children (546 boys and 389 girls) were included in this analysis, aged 7–12 years. The detailed characteristics of normal weight participants and those with overweight/obesity are presented in Table 1. Compared with normal weight subjects, children with overweight and obesity were older; were mostly urban children; had a higher BMI, heart rate, SBP, DBP, PP, and MAP; and had a higher body fat, muscle mass, protein mass, FAT%, visceral fat area, and visceral fat level (*p* < 0.05). Compared with boys, girls with overweight and obesity had a higher heart rate, body fat, and FAT%, and a lower SBP, PP, muscle mass, protein mass, visceral fat area, and visceral fat level (*p* < 0.05).

**Table 1:** Blood pressure-related indexes and body composition characteristics of school-age children (*n* = 935).

**Variables**	**Normal weight (*n* = 307)**	**Overweight/obesity (*n* = 628)**	***P*-value**	**Overweight/obesity**		***P*-value**
				**Boys (*n* = 378)**	**Girls (*n =* 250)**	
Setting			0.002			0.229
Urban	140 (45.60)	355 (56.53)		221 (58.47)	134 (53.60)	
Rural	167 (54.40)	273 (43.47)		157 (41.53)	116 (46.40)	
Age (years)	9.17 (8.53–10.85)	10.00 (8.90–11.21)	<0.0001	10.13 (8.82–11.35)	9.79 (8.95–11.01)	0.124
Height (cm)	138.51 ± 9.95	144.97 ± 9.38	<0.0001	145.22 ± 9.28	144.60 ± 9.54	0.420
BMI (kg/m^2^)	17.40 (16.30–18.60)	23.00 (20.90–25.40)	<0.0001	23.40 (21.18–25.60)	22.40 (20.70–25.10)	0.057
Heart rate (bpm)	95 ± 13	100 ± 14	<0.0001	99 ± 14	102 ± 13	0.003
SBP (mm Hg)	94.33 (89.00–101.67)	104.67 (97.67–112.50)	<0.0001	106.38 ± 10.82	103.91 ± 12.1	0.008
DBP (mm Hg)	63.67 (59.00–69.33)	69.00 (62.67–75.67)	<0.0001	70.11 ± 9.24	69.72 ± 9.66	0.606
PP (mm Hg)	31.33 (26.00–35.33)	34.67 (29.33–41.17)	<0.0001	36.26 ± 8.62	34.19 ± 9.27	0.004
MAP (mm Hg)	74.70 ± 7.80	81.77 ± 9.21	<0.0001	82.20 ± 8.91	81.12 ± 9.62	0.147
Body fat (kg)	4.40 (2.85–5.90)	11.60 (8.10–15.40)	<0.0001	11.00 (7.10–14.43)	12.70 (2.30–3.20)	<0.0001
Muscle mass (kg)	27.36 ± 5.76	34.55 ± 6.40	<0.0001	35.96 ± 6.17	32.41 ± 6.15	<0.0001
Protein (kg)	6.27 ± 1.42	7.60 ± 1.34	<0.0001	7.98 ± 1.24	7.03 ± 1.27	<0.0001
FAT (%)	13.40 ± 5.84	23.87 ± 5.94	<0.0001	21.90 (17.15–25.90)	26.80 (24.00–30.80)	<0.0001
Visceral fat area (cm^2^)	25.00 (23.00–30.00)	58.00 (38.75–72.00)	<0.0001	69.00 (55.00–80.00)	37.00 (29.00–54.75)	<0.0001
Visceral fat level^#^			<0.0001			<0.0001
Subcutaneous/balanced	293 (100.00)	477 (76.81)		267 (71.39)	210 (85.02)	
Critical visceral obesity	0	90 (14.49)		62 (16.58)	28 (11.34)	
Visceral obesity	0	54 (8.70)		45 (12.03)	9 (3.64)	

*Data are median (interquartile range) or no. (%)*.

# *Partial data deletion*.

*BMI, body mass index; SBP, systolic blood pressure; DBP, diastolic blood pressure; PP, pulse pressure; MAP, mean arterial pressure; FAT%, body fat percentage*.

*t–test was used to explore the difference of between groups of continuous variables in normal distribution. Mann–Whitney Wilcoxon test was used to explore the difference of between groups of continuous variables in non–normal distribution. Pearson's chi–square was used to compare differences among the category data*.

### The Distribution of Pre-EBP/EBP, EPP, and EMAP in Children With Overweight and Obesity

Compared with normal BMI children, participants with overweight/obesity had higher pre-EBP/EBP, EPP, and EMAP proportions (*p* < 0.05), especially for boys. In addition, as BMI increased in boys, the proportion of normal BP, PP, and MAP decreased, and the proportion of pre-EBP/EBP, EPP, and EMAP increased. Among girls, only BP and MAP indicated the same trend (Supplementary Table 1). In particular, with an increase of BMI, BP showed a linear increasing trend in both boys and girls (*p* < 0.05). Similarly, compared with children of normal FAT% and visceral fat levels, participants with overweight and obesity had higher pre-EBP/EBP, EPP, and EMAP proportions (Supplementary Tables 2, 3). Moreover, FAT% and visceral fat levels seem to be more sensitive than BMI; with an increase of FAT% and visceral fat levels, the proportion of EPP in girls with overweight and obesity also showed an increasing trend.

### Adjusted Population Attributable Risk of Overweight/Obesity for Pre-EBP/EBP, EPP, and EMAP

The associations of overweight and obesity with pre-EBP/EBP, EPP, and EMAP were evaluated by multivariate logistic regression analysis. In boys, overweight and obesity were associated with pre-EBP/EBP, EPP, and EMAP after adjusting for age, setting, height, and heart rate. Especially as BMI increased, this association gradually increased. However, overweight and obesity were only associated with pre-EBP/EBP and EMAP of school-age girls. Because the EPP group of girls was not accompanied by visceral obesity, logistic regression analysis did not show results (Table 2).

**Table 2:** Multivariate Logistic regression analysis of the relationship of overweight/obesity with elevated BP, PP, and MAP.

	**Pre–EBP/EBP**		**EPP**		**EMAP**	
	**OR (95%CI) ^**#**^**	***P*-value^**#**^**	**OR (95%CI)^**#**^**	***P*-value^**#**^**	**OR (95%CI)^**#**^**	***P*-value^**#**^**
Boys						
Normal BMI	1.00		1.00		1.00	
Overweight	1.99 (1.13–3.50)	0.017	2.04 (0.79–5.24)	0.139	2.71 (0.70–10.48)	0.148
Obesity	4.77 (2.84–8.04)	<0.0001	3.08 (1.28–7.41)	0.012	6.66 (1.96–22.71)	0.002
Normal FAT%	1.00		1.00		1.00	
Mildly elevated FAT%	1.95 (1.20–3.18)	0.007	2.61 (1.31–5.22)	0.007	2.05 (0.88–4.81)	0.098
Moderate/severe elevated FAT%	4.83 (2.82–8.28)	<0.0001	1.94 (0.91–4.16)	0.089	4.38 (1.95–9.84)	<0.0001
Normal visceral fat level	1.00		1.00		1.00	
Critical visceral obesity	3.45 (1.91–6.22)	<0.0001	0.93 (0.39–2.17)	0.858	1.92 (0.85–4.35)	0.116
Visceral obesity	4.37 (2.13–8.97)	<0.0001	1.53 (0.65–3.56)	0.329	4.07 (1.78–9.33)	0.001
Girls						
Normal BMI	1.00		1.00		1.00	
Overweight	1.80 (0.97–3.36)	0.062	1.74 (0.50–6.03)	0.380	2.99 (0.87–10.32)	0.083
Obesity	2.35 (1.37–4.06)	0.002	2.30 (0.79–6.71)	0.128	4.74 (1.56–14.38)	0.006
Normal FAT%	1.00		1.00		1.00	
Mildly elevated FAT%	1.64 (0.96–2.81)	0.072	1.20 (0.44–3.30)	0.726	0.79 (0.29–2.12)	0.638
Moderate/severe elevated FAT%	1.85 (1.01–3.38)	0.046	1.39 (0.53–3.67)	0.503	2.62 (1.15–5.97)	0.022
Normal visceral fat level	1.00		1.00		1.00	
Critical visceral obesity	1.56 (0.69–3.62)	0.284	1.66 (0.57–4.81)	0.354	3.89 (1.51–10.02)	0.005
Visceral obesity	5.87 (1.14–30.16)	0.034	—	—	1.82 (0.34–9.81)	0.487

*EBP, elevated blood pressure; EPP, elevated pulse pressure; EMAP, elevated mean arterial pressure*.

*Data are odds ratio (ORs) and 95% confidence intervals (95% CIs)*.

# *Multivariate logistic regression analysis with the adjustment of age, setting, height and heart rate*.

The PAR of pre-EBP/EBP and EMAP attributable to overweight and obesity was greater than that of EPP. Moreover, for boys, overweight and obesity accounted for more PAR for pre-EBP/EBP, EPP, and EMAP. Pre-EBP/EBP in about 79% of boys and 76% of girls could be attributed to visceral fat level, and pre-EBP/EBP in about 76% of boys and 65% of girls could be attributed to BMI. Visceral fat level accounted for PAR of pre-EBP/EBP more than BMI. However, BMI was superior to FAT% and visceral fat level in estimating the PAR of EPP and EMAP (Table 3).

**Table 3:** Adjusted population attributable risk of overweight/obesity for elevated BP, PP, and MAP.

	**No. of** ** pre–EBP/EBP**	**No. of** ** overweight**	**No. of** ** obesity**	**PAR%**	**No. of** ** EPP**	**No. of** ** overweight**	**No. of** ** obesity**	**PAR%**	**No. of** ** EMAP**	**No. of** ** overweight**	**No. of** ** obesity**	**PAR%**
Boys												
BMI				76				66				82
FAT%	194	41	126	76	66	17	42	57	53	9	41	74
Visceral fat level				79				30				72
Girls												
BMI				65				61				78
FAT%	135	31	72	57	30	7	18	38	40	9	27	45
Visceral fat level				76				—				57

*Population attributable risk analysis adjusting for age, setting, height and heart rate*.

*EBP, elevated blood pressure; EPP, elevated pulse pressure; EMAP, elevated mean arterial pressure; PAR, population-attributable risk*.

### Correlation of Overweight and Obesity With BP, PP, and MAP

The BMI, FAT%, and visceral fat area of school-age children strongly correlated with SBP and MAP, especially for boys, and the correlation coefficients *r* > 0.5. Moreover, BMI and visceral fat area were consistent, and both showed strong correlation with BP, PP, and MAP (Supplementary Table 4).

### The Effects of Obesity on SBP, DBP, PP, and MAP With Different Height and Age

**Figure 1:**
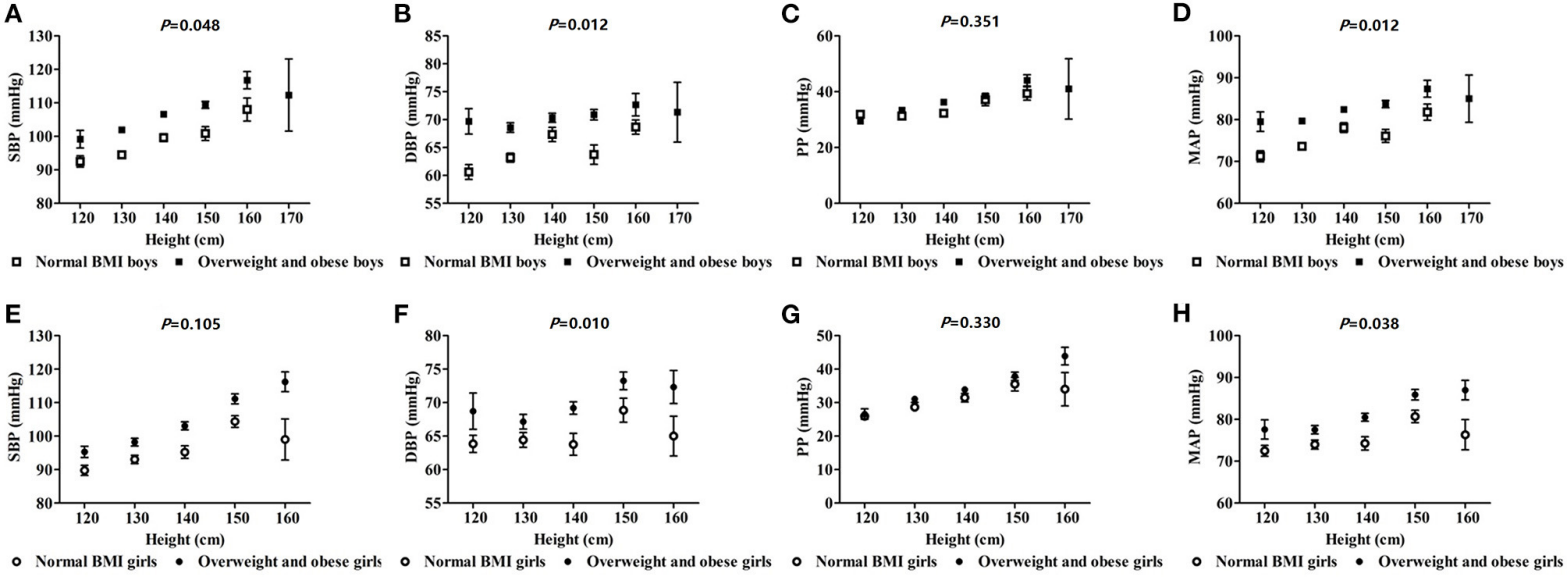
The effect of interactions of height and obesity on blood pressure in school-age children.

**Figure 2:**
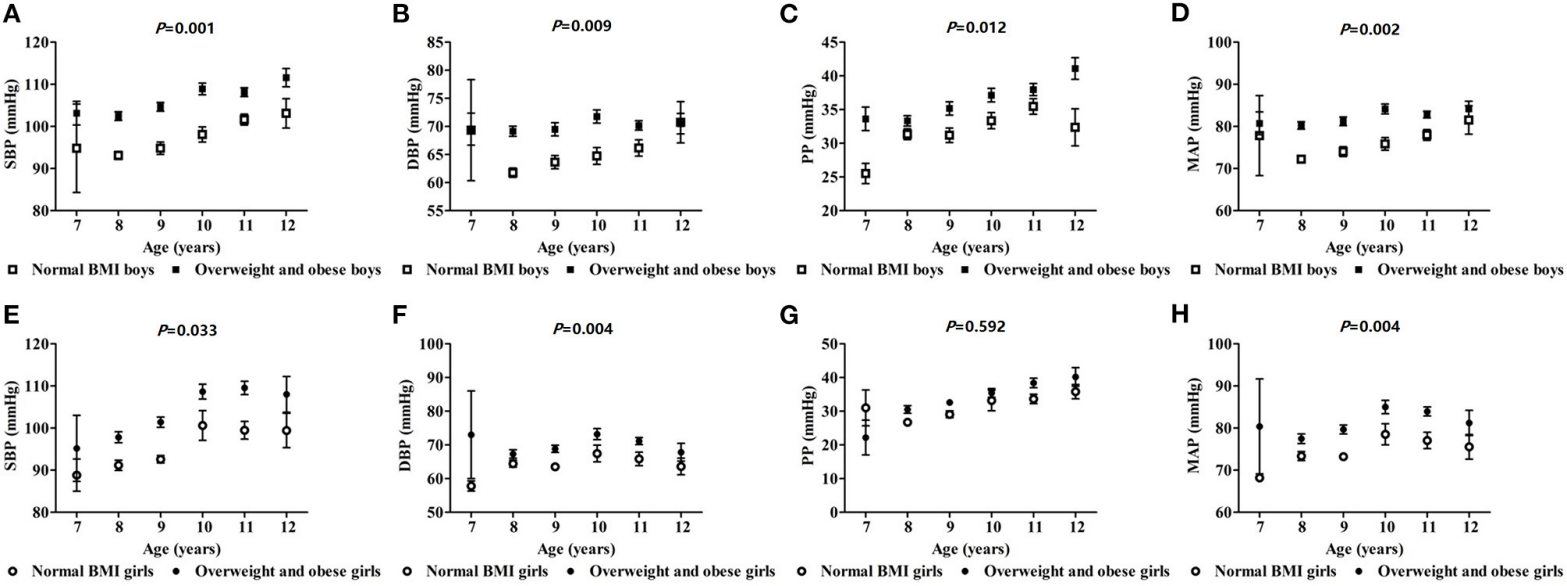
The effect of interactions of age and obesity on blood pressure in school-age children.

SBP, DBP, and MAP increased with height in boys, and were higher than those of the normal BMI group in the overweight and obesity group (*p* < 0.05). However, only DBP and MAP showed the same trends among girls. So, the relationship between blood pressure and height was augmented comparably in boys with overweight and obesity (based on BMI) (Figure 1). In boys, SBP, DBP, PP, and MAP increased with age and those values in the overweight and obesity group were higher than those of the normal BMI group (*p* < 0.05). In girls, the same trend was seen for SBP, DBP, and PP. Similarly, the interaction effect of blood pressure and age was more pronounced in boys with overweight and obesity (Figure 2).

## Discussion

The objectives of this study were to determine if there was a sex difference in the effect of being overweight or obese on blood pressure in children and to explore the impact of being overweight or obese on pulse pressure and mean arterial pressure in children differentiated by sex. We found that boys with overweight and obesity were more likely to have higher pre-EBP/EBP, EPP, and EMAP, and overweight and obesity had a greater impact on BP and MAP than PP, especially in boys. Body fat percentage and visceral fat level were better indicators to evaluate the effect of obesity on blood pressure than BMI in girls. The interaction between blood pressure and height or age was moderately increased in children with overweight and obesity, especially in boys.

EBP in children and adolescents is a growing health problem, along with the worldwide increase in obesity. Primary hypertension in children is associated with other risk factors for cardiovascular disease (CVD), including hyperlipidemia and insulin resistance (2). Children may also experience damage to organs from hypertension, including left ventricular hypertrophy and pathological vascular changes (16). PP and MAP increase with age. EBP, EPP, and EMAP can cause loss of vascular elasticity, leading to vascular stiffness in adults, which predicts incident CVD events. PP is determined by large artery stiffness and flow pulsatility, whereas MAP is determined by small resistance arterial function and cardiac output, which may provide additional insight into the underlying pathophysiology of elevated BP (17). The effect of obesity on MAP is greater than PP in our study, indicating that childhood obesity can lead to increased vascular resistance of small arteries rather than stiffness of large arteries.

High fat mass and high body fat percentage were observed to be associated with a high risk of childhood hypertension (18, 19). Hypertension in children was not only related to excessive fat in the body, but also related to the distribution of fat. Visceral adipose tissue was considered to be a kind of “ectopic fat” because this fat reservoir was not the usual location of fat storage, compared with subcutaneous adipose tissue. In particular, abdominal visceral adipose tissue was more likely to increase the risk of CVD (20, 21). A pediatric study has found a significant association between fat masses (especially intra-abdominal fat) and arterial wall thickness and stiffness in children (22). Boys and girls have different developmental characteristics of adipose tissue and non-adipose tissue. BMI growth curve and body fat development characteristics are not completely consistent, and there are gender differences (23). The body fat mass of boys increases year by year from the age of 6 to 12 and begins to decrease at the age of 13. However, the increased body fat of girls occurs early in life and is maintained during adolescence, which may be related to the increase of estrogen during puberty (24, 25). Therefore, this study uses BMI, body fat percentage, and visceral fat area or level to simultaneously assess the effect of obesity on blood pressure in order to obtain more accurate research results.

Our research results suggest that obesity is associated with elevated blood pressure through changes at the small and large artery levels, especially changes in small arteries, which was more pronounced in boys. Several studies have suggested that arterial hypertension may originate early in life, and changes in microvascular structure represent a potential mechanism for the development of hypertension (26, 27). Persistent obesity and elevated blood pressure may cause structural remodeling of the microvascular and macrovascular wall, which may further trigger arteriolar narrowing and increased large artery stiffness (28). Other studies have provided evidence that obesity-induced hypertension occurs *via* distinct pathways for men and women. For example, men develop overt CVD at an earlier age compared with women. This sex disparity continues until women reach menopause, at which time the incidence of new CVD in women climbs rapidly until it outpaces that of men. This change is due to the loss of estrogen's cardioprotective effect (29). Thus, the sex differences in the effects of obesity on large and small arteries may also be one of the reasons why CVD occurs more frequently in men than in women.

The average blood pressure of children gradually increases with age. The trend of increasing blood pressure in boys and girls is that SBP is greater than DBP, but the growth rate of boys is greater than that of girls. Therefore, the phenomenon that blood pressure rises with age is mainly related to growth and development, and is a concomitant phenomenon of growth and development of children. We found that the interaction between blood pressure and height or age in children with overweight and obesity was increased, especially for boys. Our results also suggest that the correlation between obesity and SBP is higher than the correlation with DBP, possibly due to the increase in left ventricular mass and wall thickness in children with overweight and obesity (30). In addition, obesity causes metabolic changes such as insulin resistance and high free fatty acid concentrations, leading to arterial stiffness. Arterial stiffness is considered to be an important cause of systolic blood pressure variability (31, 32). Sex differences in the risk of high systolic blood pressure may be related to the effect of sex steroids on blood pressure to some extent (33). Moreover, the difference may also be related to the distribution of fat. That is, preferentially more subcutaneous and “safe” weight is gained in girls than boys (34). Males develop earlier and more severe hypertension than females (35). Therefore, early detection and management of boyhood obesity may effectively reduce the incidence of hypertension in adulthood. Healthy nutrition, physical activity, family habits, and parenting strategies are effective in preventing or treating childhood obesity (36, 37).

This study has some limitations. First, the cross-sectional design does not show a trend in the effect of obesity on elevated blood pressure in different sex. Second, the relatively small sample sizes may prevent us from estimating accurate statistical values. Finally, in pediatrics, there are no definite diagnostic criteria for EPP and EMAP. In this study, EPP and EMAP were defined as PP and MAP ≥90th percentile of data, but a large sample of people is needed to determine the diagnostic criteria.

## Conclusions

In conclusion, our study indicates that the effect of overweight and obesity on blood pressure, mean arterial pressure, and pulse pressure is greater in boys than girls, and this effect is not affected by age and height. The effect of overweight and obesity on blood pressure was mediated mainly through small rather than large arteries.

## Data Availability Statement

The original contributions presented in the study are included in the article/Supplementary Material, further inquiries can be directed to the corresponding author/s.

## Ethics Statement

The study was approved by the Ethics Committee of Henan University. Written informed consent to participate in this study was provided by the participants' legal guardian/next of kin.

## Author Contributions

LZ, HH, and SY participated in the design and the data analysis of the study. LZ and HH drafted the manuscript. NQ, YD, LQ, JL, YL, YH, ZL, and HP collected and managed data. SJ and XG reviewed and edited the manuscript. LZ supervised the research process. All authors approved the final article and approved the submitted version.

## Conflict of Interest

The authors declare that the research was conducted in the absence of any commercial or financial relationships that could be construed as a potential conflict of interest.

## Publisher's Note

All claims expressed in this article are solely those of the authors and do not necessarily represent those of their affiliated organizations, or those of the publisher, the editors and the reviewers. Any product that may be evaluated in this article, or claim that may be made by its manufacturer, is not guaranteed or endorsed by the publisher.

## Supplementary Material

Supplementary Figure 1 A flow chart of the sampling process for the study object.

Supplementary Table 1 The distribution of BP, PP, and MAP of school-age children with different BMI.

Supplementary Table 2 The distribution of BP, PP, and MAP of school-age children with different FAT%.

Supplementary Table 3 The Distribution of BP, PP, and MAP of school-age children with different visceral fat levels.

Supplementary Table 4 Correlations of overweight/obesity with BP, PP, and MAP.
