# Supplementary figures and images for: Sex-Specific Differences in Related Indicators of Blood Pressure in School-Age Children With Overweight and Obesity: A Cross-Sectional Study
Source: Front Pediatr. 2021 Aug 5;9:674504. doi: 10.3389/fped.2021.674504 (PMC8374442; doi:10.3389/fped.2021.674504)

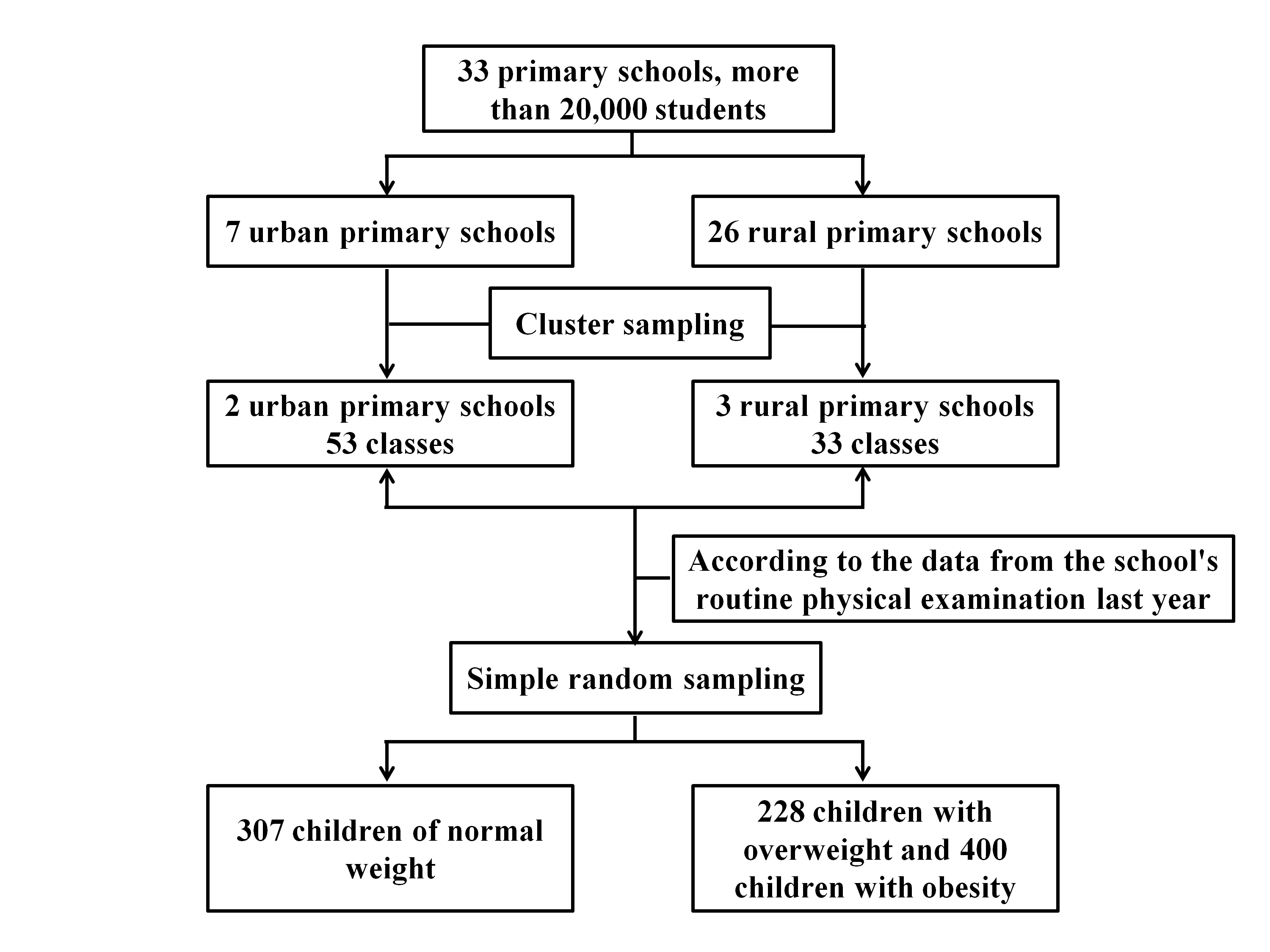

Supplement: Supplementary Figure 1 — A flow chart of the sampling process for the study object. [file Image_1.TIF]
