# Supplementary material for: Sex-Specific Differences in Related Indicators of Blood Pressure in School-Age Children With Overweight and Obesity: A Cross-Sectional Study
Source: Front Pediatr. 2021 Aug 5;9:674504. doi: 10.3389/fped.2021.674504 (PMC8374442; doi:10.3389/fped.2021.674504)
Supplement: Supplementary Table 1 — The distribution of BP, PP, and MAP of school-age children with different BMI. [file Table_1.docx]

**SUPPLEMENTARY TABLE 1** The Distribution of BP, PP and MAP of School-age Children with Different BMI

|  | Normal BMI | Overweight | Obesity | *χ*^2^ | *P* value |
| --- | --- | --- | --- | --- | --- |
| Boys |  |  |  |  |  |
| Normal BP | 141 (83.93) | 101 (71.13) | 110 (46.61) | 62.79 | <0.0001* |
| Pre-EBP | 13 (7.74) | 19 (13.38) | 41 (17.37) |  |  |
| EBP | 14 (8.33) | 22 (15.49) | 85 (36.02) |  |  |
| Normal PP | 161(95.83) | 125 (88.03) | 194 (82.20) | 17.16 | <0.0001 |
| EPP | 7 (4.17) | 17 (11.97) | 42 (17.80) |  |  |
| Normal MAP | 165 (98.21) | 133 (93.66) | 195 (82.63) | 29.69 | <0.0001 |
| EMAP | 3 (1.79) | 9 (6.34) | 41 (17.37) |  |  |
| Girls |  |  |  |  |  |
| Normal BP | 107 (76.98) | 55 (63.95) | 92 (56.10) | 15.65 | <0.0001* |
| Pre-EBP | 14 (10.07) | 10 (11.63) | 22 (13.41) |  |  |
| EBP | 18 (12.95) | 21 (24.42) | 50 (30.49) |  |  |
| Normal PP | 134 (96.40) | 79 (91.86) | 146 (89.02) | 5.78 | 0.055 |
| EPP | 5 (3.60) | 7 (8.14) | 18 (10.98) |  |  |
| Normal MAP | 135 (97.12) | 77 (89.53) | 137 (83.54) | 15.06 | 0.001 |
| EMAP | 4 (2.88) | 9 (10.47) | 27 (16.46) |  |  |

Data are no. (%).

EBP, elevated blood pressure; EPP, elevated pulse pressure; EMAP, elevated mean arterial pressure.

Pearson's chi-square and chi-square test of linear trend were used to explore the difference of between groups of category variables.

^*^ Chi-square test of linear trend.
