# Supplementary material for: Sex-Specific Differences in Related Indicators of Blood Pressure in School-Age Children With Overweight and Obesity: A Cross-Sectional Study
Source: Front Pediatr. 2021 Aug 5;9:674504. doi: 10.3389/fped.2021.674504 (PMC8374442; doi:10.3389/fped.2021.674504)
Supplement: Supplementary Table 2 — The distribution of BP, PP, and MAP of school-age children with different FAT%. [file Table_2.docx]

**SUPPLEMENTARY TABLE 2** The Distribution of BP, PP and MAP of School-age Children with Different FAT%

|  | Normal FAT% | Mildly elevated FAT% | Moderate/severe elevated FAT% | *χ*^2^ | *P* value |
| --- | --- | --- | --- | --- | --- |
| Boys |  |  |  |  |  |
| Normal BP | 232 (76.07) | 71 (60.17) | 42 (37.17) | 58.94 | <0.0001* |
| Pre-EBP | 33 (10.82) | 17 (14.41) | 21 (18.58) |  |  |
| EBP | 40 (13.11) | 30 (25.42) | 50 (44.25) |  |  |
| Normal PP | 286 (93.77) | 94 (79.66) | 91 (80.53) | 23.14 | <0.0001 |
| EPP | 19 (6.23) | 24 (20.34) | 22 (19.47) |  |  |
| Normal MAP | 293 (96.07) | 105 (88.98) | 85 (75.22) | 40.42 | <0.0001 |
| EMAP | 12 (3.93) | 13 (11.02) | 28 (24.78) |  |  |
| Girls |  |  |  |  |  |
| Normal BP | 155 (71.76) | 57 (60.00) | 42 (53.85) | 11.47 | 0.001* |
| Pre-EBP | 24 (11.11) | 13 (13.68) | 9 (11.54) |  |  |
| EBP | 37 (17.13) | 25 (26.32) | 27 (34.62) |  |  |
| Normal PP | 206 (95.37) | 86 (90.53) | 67 (85.90) | 7.77 | 0.021 |
| EPP | 10 (4.63) | 9 (9.47) | 11 (14.10) |  |  |
| Normal MAP | 202 (93.52) | 88 (92.63) | 59 (75.64) | 21.01 | <0.0001 |
| EMAP | 14 (6.48) | 7 (7.37) | 19 (24.36) |  |  |

Data are no. (%).

EBP, elevated blood pressure; EPP, elevated pulse pressure; EMAP, elevated mean arterial pressure.

Pearson's chi-square and chi-square test of linear trend were used to explore the difference of between groups of category variables.

^*^ Chi-square test of linear trend.
