# Supplementary material for: Sex-Specific Differences in Related Indicators of Blood Pressure in School-Age Children With Overweight and Obesity: A Cross-Sectional Study
Source: Front Pediatr. 2021 Aug 5;9:674504. doi: 10.3389/fped.2021.674504 (PMC8374442; doi:10.3389/fped.2021.674504)
Supplement: Supplementary Table 3 — The Distribution of BP, PP, and MAP of school-age children with different visceral fat levels. [file Table_3.docx]

**SUPPLEMENTARY TABLE 3** The Distribution of BP, PP and MAP of School-age Children with Different Visceral Fat Levels

|  | Normal visceral fat level | Critical visceral obesity | Visceral obesity | *χ*^2^ | *P* value |
| --- | --- | --- | --- | --- | --- |
| Boys |  |  |  |  |  |
| Normal BP | 302 (71.23) | 24 (38.71) | 14 (31.11) | 46.30 | <0.0001* |
| Pre-EBP | 50 (11.79) | 11 (17.74) | 10 (22.22) |  |  |
| EBP | 72 (16.98) | 27 (43.55) | 21 (46.67) |  |  |
| Normal PP | 381 (89.86) | 53 (85.48) | 33 (73.33) | 10.88 | 0.004 |
| EPP | 43 (10.14) | 9 (14.52) | 12 (26.67) |  |  |
| Normal MAP | 397 (93.63) | 51 (82.26) | 30 (66.67) | 37.63 | <0.0001 |
| EMAP | 27 (6.37) | 11 (17.74) | 15 (33.33) |  |  |
| Girls |  |  |  |  |  |
| Normal BP | 235 (67.92) | 15 (53.57) | 2 (22.22) | 4.99 | 0.025* |
| Pre-EBP | 40 (11.56) | 3 (10.71) | 2 (22.22) |  |  |
| EBP | 71 (20.52) | 10 (35.71) | 5 (55.56) |  |  |
| Normal PP | 322 (93.06) | 22 (78.57) | 9 (100.00) | — | 0.039^#^ |
| EPP | 24 (6.94) | 6 (21.43) | 0 |  |  |
| Normal MAP | 320 (92.49) | 19 (67.86) | 7 (77.78) | — | <0.0001^#^ |
| EMAP | 26 (7.51) | 9 (32.14) | 2 (22.22) |  |  |

Data are no. (%).

EBP, elevated blood pressure; EPP, elevated pulse pressure; EMAP, elevated mean arterial pressure.

Pearson's chi-square, Fisher's exact test and chi-square test of linear trend were used to explore the difference of between groups of category variables.

^*^ Chi-square test of linear trend.

^#^ Fisher's exact test.
