# Supplementary material for: Sex-Specific Differences in Related Indicators of Blood Pressure in School-Age Children With Overweight and Obesity: A Cross-Sectional Study
Source: Front Pediatr. 2021 Aug 5;9:674504. doi: 10.3389/fped.2021.674504 (PMC8374442; doi:10.3389/fped.2021.674504)
Supplement: Supplementary Table 4 — Correlations of overweight/obesity with BP, PP, and MAP. [file Table_4.docx]

**SUPPLEMENTARY TABLE 4** Correlations of Overweight/obesity with BP, PP and MAP

|  | SBP | DBP | PP | MAP |
| --- | --- | --- | --- | --- |
| Boys |  |  |  |  |
| BMI | 0.53, <0.0001 | 0.41, <0.0001 | 0.26, <0.0001 | 0.50, <0.0001 |
| FAT% | 0.53^*^, <0.0001 | 0.43, <0.0001 | 0.23, <0.0001 | 0.51^*^, <0.0001 |
| Visceral fat area | 0.53, <0.0001 | 0.44, <0.0001 | 0.23, <0.0001 | 0.51, <0.0001 |
| Girls |  |  |  |  |
| BMI | 0.47^*^, <0.0001 | 0.28^*^, <0.0001 | 0.36^*^, <0.0001 | 0.38^*^, <0.0001 |
| FAT% | 0.45^*^, <0.0001 | 0.33^*^, <0.0001 | 0.28^*^, <0.0001 | 0.41^*^, <0.0001 |
| Visceral fat area | 0.48, <0.0001 | 0.32, <0.0001 | 0.32, <0.0001 | 0.42, <0.0001 |

Spearman’s rank correlation test was used for continuous variable of non-normal distribution and Pearson's correlation test was used for continuous variable of normal distribution.

^*^Pearson correlation coefficient.

FAT%, body fat percentage.
